# Human Capital and Employment Outcomes Among Foreign Educated and US Nurses Working in Long Term Care

**DOI:** 10.1093/geroni/igab046.3189

**Published:** 2021-12-17

**Authors:** Roy Thompson, Susan Silva, Kirsten Corazzini, Thomas Konrad, Michael Cary, Eleanor McConnell

**Affiliations:** 1 Duke University, Durham, North Carolina, United States; 2 University of Maryland School of Nursing, Baltimore, Maryland, United States; 3 University of North Carolina at Chapel Hill, Chapel Hill, North Carolina, United States; 4 Duke University School of Nursing, Durham, North Carolina, United States

## Abstract

Employing Foreign Educated Nurses (FENs) helps address Registered Nurse (RN) shortages in long-term care (LTC) in the United States (US). However, examination of factors explaining differences in their employment outcomes relative to US Educated Nurses (USENs) is limited. This study uses 2018 National Sample Survey of Registered Nurses data to compare income, work hours, job satisfaction, and human capital, defined as personal characteristics (knowledge, work experience) and behaviors (job mobility), of FENS and USENs working full-time in LTC. A human capital score, consisting of highest nursing education, skill certifications, state licensures, years of experience, multi-state employment history, and multi-lingual status was constructed. Covariates included nurse demographics, direct care role, and ability to practice to full scope. Covariate-adjusted group differences in employment outcomes and human capital were compared using ANCOVA and logistic regression. Mediation analyses explored whether human capital explained FEN vs USEN differences. FENs earned higher hourly wages (p=0.0169), worked fewer hours annually (p=0.0163), and reported greater human capital (p<.0001) compared to USENs. FENs and USENs, however, had similar annual salaries (p=0.3101) and job satisfaction (p=0.1674). Human capital mediated FEN vs USEN effects on hourly wages but not annual work hours. FENs’ higher levels of human capital partially account for FEN vs USEN differences in hourly wages. Application of the human capital concept advanced our ability to examine differences in employment outcomes and highlight aspects of the value that FENs contribute to LTC settings.

